# Mechanistic Modeling of Gene Regulation and Metabolism Identifies Potential Targets for Hepatocellular Carcinoma

**DOI:** 10.3389/fgene.2020.595242

**Published:** 2020-12-23

**Authors:** Renliang Sun, Yizhou Xu, Hang Zhang, Qiangzhen Yang, Ke Wang, Yongyong Shi, Zhuo Wang

**Affiliations:** Bio-X Institutes, Key Laboratory for the Genetics of Developmental Neuropsychiatric Disorders (Ministry of Education), Shanghai Jiao Tong University, Shanghai, China

**Keywords:** regulatory-metabolic integration, metabolic model, molecular stratification, potential therapeutic target, hepatocellular carcinoma, metabolic reprogramming

## Abstract

Hepatocellular carcinoma (HCC) is the predominant form of liver cancer and has long been among the top three cancers that cause the most deaths worldwide. Therapeutic options for HCC are limited due to the pronounced tumor heterogeneity. Thus, there is a critical need to study HCC from a systems point of view to discover effective therapeutic targets, such as through the systematic study of disease perturbation in both regulation and metabolism using a unified model. Such integration makes sense for cancers as it links one of the dominant physiological features of cancers (growth, which is driven by metabolic networks) with the primary available omics data source, transcriptomics (which is systematically integrated with metabolism through the regulatory-metabolic network model). Here, we developed an integrated transcriptional regulatory-metabolic model for HCC molecular stratification and the prediction of potential therapeutic targets. To predict transcription factors (TFs) and target genes affecting tumorigenesis, we used two algorithms to reconstruct the genome-scale transcriptional regulatory networks for HCC and normal liver tissue. which were then integrated with corresponding constraint-based metabolic models. Five key TFs affecting cancer cell growth were identified. They included the regulator *CREB3L3*, which has been associated with poor prognosis. Comprehensive personalized metabolic analysis based on models generated from data of liver HCC in The Cancer Genome Atlas revealed 18 genes essential for tumorigenesis in all three subtypes of patients stratified based on the non-negative matrix factorization method and two other genes (*ACADSB* and *CMPK1*) that have been strongly correlated with lower overall survival subtype. Among these 20 genes, 11 are targeted by approved drugs for cancers or cancer-related diseases, and six other genes have corresponding drugs being evaluated experimentally or investigationally. The remaining three genes represent potential targets. We also validated the stratification and prognosis results by an independent dataset of HCC cohort samples (LIRI-JP) from the International Cancer Genome Consortium database. In addition, microRNAs targeting key TFs and genes were also involved in established cancer-related pathways. Taken together, the multi-scale regulatory-metabolic model provided a new approach to assess key mechanisms of HCC cell proliferation in the context of systems and suggested potential targets.

## Introduction

Hepatocellular carcinoma (HCC) is the most common type of primary liver cancer and is the third leading cause of cancer-related death (Ferlay et al., 2015). Obesity, diabetes, fatty liver, virus infection, and many other diseases can lead to HCC. Treatment of HCC largely depends on surgery. Radiochemotherapy is unsatisfactory in part because of the current difficulty in early diagnosis. Furthermore, although drugs like Sorafenib and Lenvatinib had been approved by the Food and Drug Administration (FDA), the drug–response rates are relatively low probably due to the pronounced tumor heterogeneity. For example, in one trial the median survival was only 2–3 months longer compared to the placebo arm in Asians and Caucasians (Cheng et al., 2009). More precise patient stratification and discovery of novel drug targets are necessary to improve treatment outcomes of HCC.

Several recent studies classified the molecular subtypes of HCC based on proteomic data. In one study, the classification of early-stage Chinese HCC samples revealed the mechanism of early tumor cell development (Jiang et al., 2019). In the other study, the classification of hepatitis B virus (HBV)-related HCC samples identified three subgroups with distinct features in metabolic reprogramming, microenvironment dysregulation, and cell proliferation (Gao et al., 2019).

Metabolic reprogramming is an important characteristic and driver of cancer. Genome-scale metabolic models (GEMs) have been successfully used to characterize cancer metabolism and to identify drug targets for cancer treatment. GEMs are a powerful framework to mechanistically represent the relationship between genotype and phenotype by computationally modeling the biochemical constraints imposed on the phenotype. The models are capable of simulating various biological tasks under given conditions (Mardinoglu and Nielsen, 2012, 2015). This allows the identification of essential genes or reactions for a particular objective function. Many disease-related genes and metabolites have been experimentally validated by comparing the altered metabolism between normal and tumor tissue models. Folger et al. (2011) used microarray data to identify key genes for non-small-cell lung cancer. Mardinoglu et al. (2014) utilized data from the Human Protein Atlas Database with the INIT algorithm to successfully construct 69 cell-specific models and 16 cancer-specific models. More recently, Uhle et al. (2017) employed RNA-Seq data from The Cancer Genome Atlas (TCGA) database together with the INIT algorithm to reconstruct 6753 patient-specific metabolic models for various cancers.

Although many anti-cancer drugs developed by target-based approaches have been approved by the FDA (Assoun et al., 2017; Howie et al., 2018), there are still few effective therapeutic targets for HCC. Bidkhori et al. (2018) recently addressed this by utilizing metabolic network topology analysis to divide 179 liver HCC (LIHC) samples from the TCGA-LIHC database into three subtypes and identify potential subtype-specific therapeutic targets. However, metabolic networks are dramatically affected by complex transcriptional regulatory networks, while the changes in transcriptional regulation can lead to changes in enzyme abundance or activity, which in turn lead to changes in physiological states (e.g., cancer cell growth). The close crosstalk between metabolic and regulatory mechanisms during the complex tumor development necessitates the investigation of multi-level mechanisms by integrating both regulation and metabolism. Since the regulatory role of miRNA in liver cancer remains largely in the work-in-progress phase, it is hard to get the full spectrum of dysregulated miRNA in HCC (Sartorius et al., 2019), we focused on the genome-scale transcriptional regulatory network between TFs and genes, which was then mechanistically combined with genome-scale liver metabolic model. Several studies are constructing global transcriptional regulatory networks for liver tissue or HCC tissue (Zhu et al., 2012; Chen et al., 2017), but to our knowledge, no computational studies have integrated regulation and metabolism into a unified genome-scale model in studying HCC.

**FIGURE 1:**
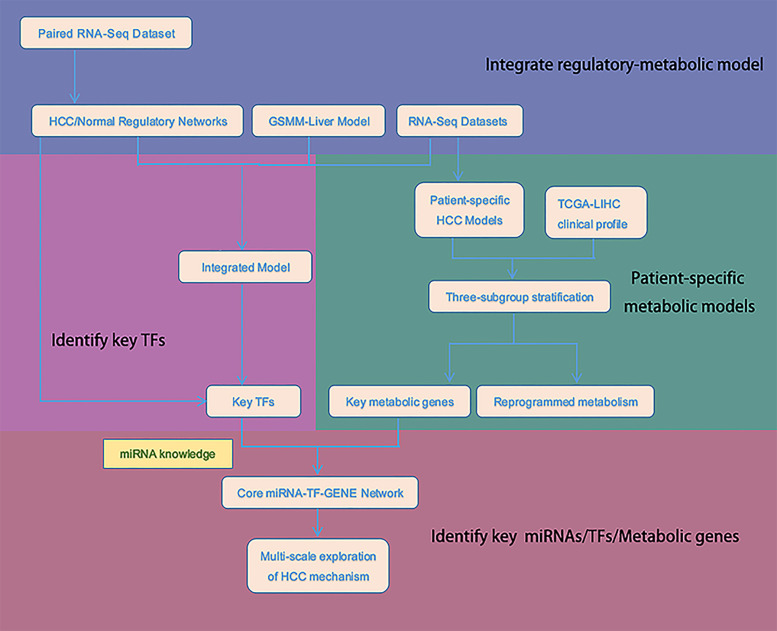
The schema of the integrated model for stratification and key targets discovery.

In this study, schematically summarized in Figure 1, we used integrated regulatory-metabolic modeling to investigate the possible mechanism of HCC using all TCGA-LIHC samples. We have previously developed the Integrated Deduced Regulation And Metabolism (IDREAM) algorithm (Wang Z. et al., 2017), which uses a bootstrapping linear regression model on large-scale gene expression datasets (e.g., 2,929 microarray for *Saccharomyces cerevisiae*) to predict TF regulation on enzyme-encoding genes, followed by a probabilistic regulation of metabolism approach to apply regulatory constraints to the metabolic network. The integrated model can predict the influence of each TF knockout on certain objective functions, such as cell growth. The model has been successfully applied in *S. cerevisiae* to effectively predict the influence of transcriptional regulation on the metabolic phenotype. It also can reveal novel synthetic lethal pairs of TFs and metabolic genes with an important interaction mechanism. But IDREAM requires a large-scale expression dataset to infer regulatory network, which is limited for HCC, so we modified it extensively for the application in liver cancer study herein. We inferred the tumor/normal regulatory networks using two independent algorithms, MERLIN and CMIP. Then The regulatory relationships deduced by both algorithms were regarded as “high confidence” regulations and were tagged in the transcriptional regulatory networks for further integration with the metabolic model. Using the integrated model, we classified HCC patients into different subgroups by expression data of transcription factors (TFs) and genes in the integrated network. The classification results were evaluated by overall survival (OS) outcomes. The integrated regulatory-metabolic model allows the identification of the mechanisms of HCC tumor cell progression, the genes associated with poor prognosis, and potential therapeutic targets. In addition, microRNAs (miRNAs) regulating the influential TFs and metabolic genes were incorporated to validate whether the genes identified by the integrated model were important for HCC tumorigenesis and their value as targets for clinical treatment. The results were consistent with previous *in-silico* and experimental studies.

## Materials and Methods

### HCC Gene Expression Data

RNA-Seq expression data were obtained from 315 HCC samples with clinical outcomes from the TCGA-LIHC Project, 232 HCC samples with clinical outcomes from the International Cancer Genome Consortium-Liver Cancer RIKEN (ICGC-LIRI) Project, and 50 HCC paired tumor-normal samples from the Gene Expression Omnibus (GEO) database (GSE77314) (Liu G. et al., 2016). The three gene expression datasets were, respectively, employed to construct the integrated regulatory-metabolic network model. The GSE77314 dataset was also used to infer tumor and normal liver regulatory networks.

### Metabolic Network Models

The genome-scale metabolic model of liver tissue used for integration was retrieved from the Human metabolic Atlas (HMA) Database (the^1^). It was built based on the combination of the HMR2 model with RNA-Seq data of liver tissue to provide an approach to explore metabolic and proteomic functions in cancer (Uhlén et al., 2015). The patient-specific GEMs of HCC used for metabolic analyses were retrieved from the BioModels Database^2^. Uhle et al. (2017) utilized the tINIT algorithm to perform the reconstruction. The characteristics of the metabolic pathways in each model were determined by the protein-coding genes expression level detected from individual patient RNA-Seq data in the TCGA-LIHC Project. Biomass representing cell growth (whose formula was also obtained from Uhle et al., 2017) was set to be the objective function. We selected 315 of 338 HCC individual models with clear clinical stage information (excluding “not reported”) for metabolic reprogramming analysis.

### Construction of Regulatory Networks

Two independent algorithms—the modular regulatory network learning with per-gene information (MERLIN) (Roy et al., 2013) and conditional mutual information measurement using a parallel computing framework CMIP (Zheng et al., 2016)—were used to construct the tumor/normal regulatory networks from the expression data (GSE77314), which were implemented using the Part 1 script in Supplementary File 1. MERLIN combines the per-gene method and per-module concept based on a probabilistic graphical model to infer regulatory network. Thus, MERLIN cannot include only memberships deduced from individual genes. The algorithm must also take the similarity within a group of genes into consideration. The algorithm is effective in predicting transcriptional changes in human differentiation neural progenitor cells (Roy et al., 2013). In addition, MERLIN outperforms several other state-of-the-art algorithms. We used default settings, except for the use of five-fold cross-validation.

The CMIP algorithm quantifies the interactions between genes on the basis of conditional mutual information measurement to avoid neglecting subtle relations under certain conditions. For example, if both A and B are strongly connected to C, then the actual relationship between A and B may be confusing because of the interference of C. The performance was evaluated by the average Area Under Curve (AUC) of 10 benchmark datasets provided by the DREAM3 algorithm. CMIP performed better than the other algorithms. Additionally, parallelized computation enabled it to handle genome-scale datasets and to complete tasks within a relatively short time compared to other popular methods presented in DREAM3 Projects (Marbach et al., 2009). CMIP was run using default parameters to let the algorithm automatically decide the threshold of the dynamic removal of gene-pairs.

The regulatory relationships deduced by both algorithms were regarded as “high confidence” regulations and were tagged in the regulatory networks for further integration with the metabolic model.

### Metabolic Analysis

The COBRA Toolbox incorporated in MATLAB was used for the metabolic analysis (Heirendt et al., 2019). Flux Balance Analysis predicts feasible phenotypic states by setting appropriate constraints gained from prior knowledge or assigned conditions. By identifying the metabolic task to be studied, the flux distribution of all reactions in the model can be calculated and solved as follows:

$$\begin{array}{cc} \mathrm{maximum}: & \text{Cell growth}\\ \mathrm{subject}\mathrm{to}: & \text{S}\cdot \text{v}=0\\ & {\text{a}}_{\text{j}}\leq \text{v}\leq {\text{b}}_{\text{j}} \end{array}$$

where v is a flux vector representing a particular flux configuration, S is the stoichiometric matrix, and a*_*j*_* and b*_*j*_* are the minimum and maximum fluxes, respectively, through reaction *j*.

We mainly used the “SingleGeneDeletion” function to find metabolic genes whose knockout led to decreased cell growth. The “OptimizeCbModel” function was used to calculate the optimal growth rate and corresponding flux distribution.

### Integration of Regulatory Network and Metabolic Model for HCC

Modeling the regulatory networks of HCC and normal liver tissue required the determination of TFs functioning in liver tissue. To do this, we used liver regulatory network information from RegulatoryCircuits (Daniel et al., 2016)^3^, which was inferred based on the interactions of TFs-promoters, TFs-enhancers, promoters-genes, and enhancers-genes. The data were validated by introducing ChIP-Seq, expression quantitative trait loci (eQTL), and RNA-Seq data. We also used the human regulatory network from the RegNetwork (Zhi-Ping et al., 2015)^4^, which was constructed by considering prior knowledge of TF binding sites and post-transcriptional regulation by miRNAs. In addition, convincing published results were also included.

The union of these two public human regulatory networks yielded 1,366 TFs. We used these 1,366 TFs along with the 2,456 metabolic genes contained in the liver tissue model in the HMA database together with GSE77314 RNA-Seq expression data to determine the regulatory associations in the HCC and normal liver metabolic models. Different from the bootstrapping linear regression model used for regulatory associations inference in IDREAM, here we applied two independent algorithms, MERLIN and CMIP to calculate the interactions. The union of the results predicted by the two methods represented the regulatory network. The overlapping interactions represented ‘high confidence’ interactions. Then we used the probabilistic regulation of metabolism approach to build the integrated regulatory-metabolic model and predicted TFs affecting cell growth in tumor and normal liver. We first calculated the probability of a target gene being ON when TF was OFF, designated as Prob(Gene = ON| Factor = OFF). The constraints on the corresponding reaction flux were *V*_*max*_ × Prob, where Vmax was derived by flux variability analyses. We then simulated the changes in cell growth and each reaction flux. The implementation of the integrated model construction code by MATLAB is provided in Part 2 of Supplementary File 1.

### Stratification, Survival, and Analysis of Differentially Expressed Genes (DEGs)

In total, there are 3,492 expressed genes in the integrated regulatory-metabolic network (1,366 TFs and 2,456 metabolic genes), excluding overlapping genes and those with no expression data. The expression data of these 3,492 genes were used to stratify 315 TCGA-LIHC samples using the non-negative matrix factorization (NMF) consensus clustering method from the “NMF” R package (Attila and Mattias, 2008). This machine-learning algorithm aims to distinguish different molecular patterns in high-throughput genomic data. We used 200 iterations to determine the best clustering number between two and 10? and selected the three best-value clusters according to the cophenetic correlation coefficient and average silhouette width.

Clinical outcomes of the TCGA samples were used to evaluate the clustering results. The Kaplan-Meier survival curve implemented in the “survival” R package was applied to assess the OS rate. The NMF clustering subtypes showed significant differences in survival outcomes.

For the analysis of DEGs, we used a linear model and moderated *t*-statistics based algorithm implemented in the “Limma” package, with absolute value log2(fold change) ≥1 and *P* ≤ 0.05. We compared the three clusters in pairs and selected the intersection of DEGs between Class2:Class1 and Class2:Class3 as the significantly upregulated/downregulated genes of the subtype with the worst prognosis.

Functional enrichment analyses of the Kyoto Encyclopedia of Genes and Genomes (KEGG) pathways were performed using Database for Annotation, Visualization, and Integrated Discovery (DAVID;^5^). Adjusted *P* ≤ 0.05 indicated significant enrichment.

### Network Topology Analysis

Cytoscape software was used for topology explorations (Su et al., 2014). The “Tools”–“Merge”–“networks” function with the optional parameter “difference” was used to detect differences between tumor and normal liver networks. The principle was to remove all identical nodes to identify TFs/metabolic genes that were present only in HCC or the normal regulatory network. We highlighted the hub genes being responsible for the abnormity on the topological structure.

## Results

### Differences of Regulatory Networks Between Tumor and Normal Liver Cells

**FIGURE 2:**
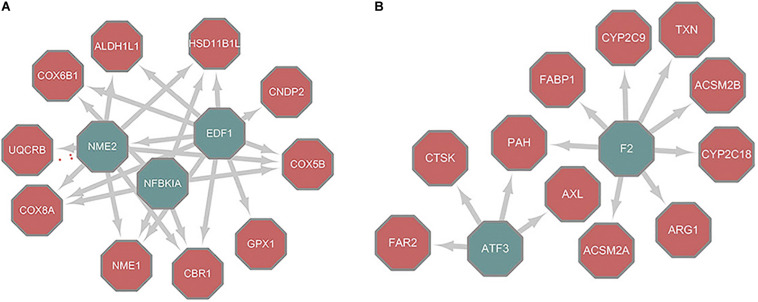
Core structure of different nodes between topology of normal/tumor regulatory networks. **(A)** Differences between normal and tumor networks. **(B)** Differences between tumor and normal networks; Nodes filled with green are TFs while nodes filled with red are metabolic genes.

There are many algorithms designed to infer regulatory networks from transcriptome profiles. The results have been validated in model organisms that include *S. cerevisiae* and *Escherichia coli*. We used the MERLIN and CMIP algorithms together with paired RNA-Seq data obtained from the GEO database (GSE77314) (Wang Z. et al., 2017) to construct the regulatory networks of HCC and paired normal tissue, implemented by the Part1 script in Supplementary File 1. There were a total of 15,143 pairs and 29,127 pairs of regulation between TFs and target genes deduced from tumor and normal samples (Supplementary Table 1). Of these, 1,654 pairs were the same. Cytoscape was used to visualize the topology difference between these two networks. After removing the nodes that had little influence, the core structure was obtained (Figure 2). In the core structure, *NME2* and *NFKBIA* were the hub TFs that were important in normal liver models (Figure 2A). These two TFs were absent in the HCC tumor model (Figure 2B). Nuclear factor κB (NF-κB) affects multiple biological processes by regulating the immune response and inflammation. NF-κB is a hallmark in cancer progression (Fengting et al., 2014). *NFKBIA* is a member of a cellular protein family that can mask the nuclear localization signals of NF-κB and block its binding to DNA. Because of this inhibition ability, *NFKBIA* has long been considered as a tumor suppressor (Laos et al., 2006). *NME*, which is located on chromosome 17q21, is a gene family associated with the suppression of cancer metastasis and invasion (Steeg et al., 1988). In particular, the *NME2* product inhibits metastasis of breast cancer and lung cancer (Hennessy et al., 1991; Krishna et al., 2014). Therefore, the reconstructed regulatory networks effectively revealed the critical known differences between liver cancer and normal tissues. *NME2* and *NFKBIA* represent putative tumor suppressor factors for future studies.

### Integrative Regulatory-Metabolic Network Identified Abnormality of Hippo Signaling as Key Misregulation in HCC

We integrated the regulatory network with metabolic models to identify potential TFs vital to the growth of HCC cells using the source code of Part2 in Supplementary File 1. The compositions of the integrated models for HCC and normal liver tissue are listed in Supplementary Table 1. The basic metabolism was consistent, while the TFs and target genes differed. There were 1313 and 1312 TFs in the HCC and normal model, respectively. These TFs included 33 that were HCC-specific and 32 that were specific for a normal liver.

Reactome database analysis of the 32 specific TFs (including *NME2* and *NFKBIA*) not involved in the HCC regulatory network revealed that they were enriched for the YAP1- and WWTR1 (TAZ)-stimulated gene expression pathways. They are transcriptional co-activators interacting with TEAD family genes to promote the expression of TFs critical to cell proliferation and apoptosis through the Hippo signaling pathway (Lehmann et al., 2016). The findings suggested that depletion of these 32 TFs might lead to abnormal Hippo signaling and might induce a wide range of cancers. In addition, the 33 specific TFs in the HCC integrated model were mainly enriched in cancer metabolism and transcriptional misregulation pathways.

For each TF knockout simulation, we changed the constraints on corresponding reactions according to activation/inhibition interactions and then simulated the cell growth rate to calculate the growth ratio relative to wildtype, as implemented in Part3 script in Supplementary File S1. We found TFs affecting both tumor and normal cell growth, as well as TFs that only reduced the growth of tumor cells (Supplementary Table 2). For example, disruption of SMAD2, HEY2, ELK1, and CREB3L3 was predicted to lead to >80% reduction in tumor cell growth while having no effect on normal cells. In particular, the involvement of HEY2 and SMAD2 in HBV induced HCC development was evident. The important TFs are likely to be effective targets for the inhibition of tumor cells of HCC.

### Precise Stratification of TCGA-LIHC Samples Based on Metabolic and Transcriptional Gene Expression

The identification of genes or pathways that could be valuable as targets for treatment has been a goal for a long time. Precise clinical diagnosis has been hindered by the pronounced heterogeneity of HCC. This heterogeneity partly reflects the inefficient current TNM stage classification. Molecular stratification of HCC patients and identification of corresponding therapeutic targets are current research goals. Bidkhori et al. (2018) utilized a metabolic network-based method to divide 179 TCGA-LIHC samples into three subtypes and identified their specific characteristics. Jiang et al. (2019) used proteomic data to classify HCC patients and explored the mechanism of an early-stage HCC tumor cell. Here, we used the expression data of 3,492 genes in the integrated model to stratify all the HCC samples with actual clinical survival information from the TCGA-LIHC dataset and to identify altered metabolism among different subgroups and specific characteristics of the poor prognosis subgroup.

**FIGURE 3:**
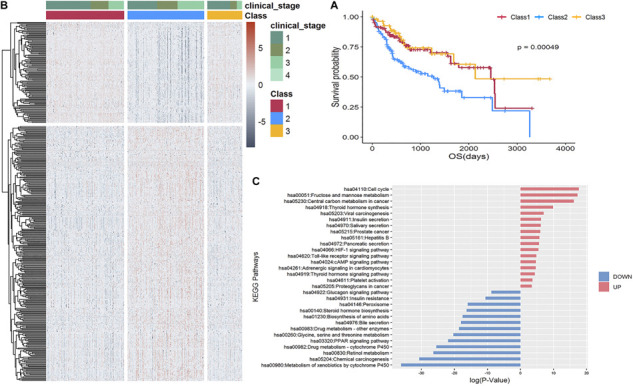
Stratification of 315 TCGA-LIHC samples. **(A)** Kaplan-Meier curve shows the survival outcomes of Class 1 (red), Class 2 (blue), and Class 3 (yellow); the *P*-value is 0.00049, which is significant. **(B)** Heatmap of 399 differentially expressed genes revealed a distinguishable pattern between Class 2 and Class 1 and 3. Red color represents upregulated while dark gray represents downregulated. **(C)** Enrichment analysis of KEGG pathways of 287 upregulated and 112 downregulated genes, respectively.

Using an NMF consensus clustering analysis, three major classes were identified in the TCGA-LIHC cohort: Class 1 (*n* = 130), Class 2 (*n* = 127), and Class 3 (*n* = 58). The survival curves (Figure 3A) revealed a significantly lower OS rate for Class 2 (*P* = 0.00049). Comparison of the 159 overlapped samples in a previous study (Bidkhori et al., 2018) and this study revealed the relatively good agreement in identifying the lowest OS subgroup: 91% (48 of 53; former results that are also in ours) and 70% (49 of 70, our results that are also in the former findings). Consequently, we focused on determining the characteristics of the Class 2 poor prognosis subgroup at the transcriptome and metabolism levels.

A supervised analysis using Limma (Matthew et al., 2015) revealed 399 differentially expressed genes having distinguishable pattern in Class 2 compared to Class 1 and 3, as shown in the heatmap in Figure 3B, comprising 287 upregulated genes [including three potential therapeutic targets: *ALDOA*, *G6PD*, and *ACSS1* specific to the lowest OS subgroup identified by Bidkhori et al. (2018)] and 112 downregulated genes (Supplementary Table 3) enriched in 17 and 13 non-overlapping KEGG pathways, respectively (Figure 3C).

**FIGURE 4:**
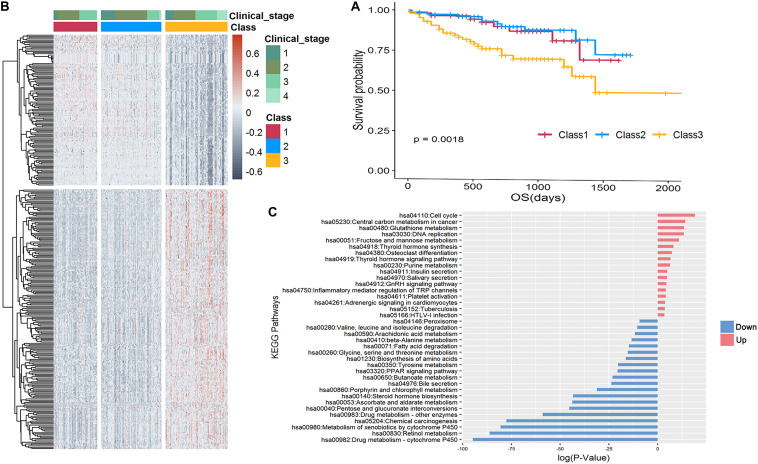
Stratification of 232 LIRI-JP samples. **(A)** Kaplan-Meier curve shows the survival outcomes of Class 1 (red), Class 2 (blue), and Class 3 (yellow); the *P*-value is 0.0018, which is significant. **(B)** Heatmap of 332 differentially expressed genes revealed a distinguishable pattern between Class 3 and Class 1 and 2. Red color represents upregulated while dark gray represents downregulated. **(C)** Enrichment analysis of KEGG pathways of 210 upregulated and 122 downregulated genes, respectively.

To validate the effectiveness of our stratification strategy, we applied the same strategy for the LIRI-JP dataset in the ICGC database to form three subgroups with significant prognosis differences (Figure 4A; *P* = 0.0018). We found 332 differentially expressed genes revealed distinguishable pattern between the poor prognosis subgroup and other two subgroups, as shown in heatmap of Figure 4B, and the pathways enriched for DEGs were very consistent with DEG-enriched pathways of TCGA-LIHC data (Figure 4C). Specifically, the upregulated genes were mainly enriched in established cancer-related pathways involved in improved cell proliferation. Notably, viral carcinogenesis and HBV pathways were upregulated and could be directly linked with HCC development. Another example is increased glucose uptake as a principal nutrient source in central carbon metabolism of cancer, cell cycle, and fructose metabolism. We also found that hypoxia-inducible factor signaling was upregulated. This signaling consists of master regulators of oxygen homeostasis that allow tumor cells to adapt to a hypoxic environment by enhancing oxygen delivery and also affect important growth factors like the *vascular endothelial growth factor* gene. In contrast, the downregulated genes were generally found in pathways contributing to drug metabolism. An example is the peroxisome proliferator-activated receptor signaling pathway, which has also been identified in less aggressive HCC subtypes through proteomics analysis, as well as drug cytochrome P450 metabolism, which is reduced in advanced cancer patients (Rivory et al., 2002).

### Phosphoinositide 3-Kinase (PI3K)-Akt and Mammalian Target of Rapamycin (mTOR) Signaling Pathways Are Critical to HCC Tumor Cell Growth

By using IDREAM, eight, 13, and five TFs were identified as being vital for HCC cell growth in Class 1, Class 2, and Class 3, respectively, of TCGA-LIHC, after excluding TFs that also affected normal tissue. Three TFs were common in all three classes (Table 1).

**TABLE 1:** HCC Cell growth ratio by influential TFs knockouts.

Ratio after knockout of common TFs in all three classes of TCGA-LIHC			
TF	Class 1	Class 2	Class 3
CTBP1	0.926	0.926	0.926
HTATIP2	0.926	0.926	0.926
ETV7	0.234	0.12	0.09
**Ratio after knockout of specific TFs in lowest survival class**			
**TF**	**TCGA-LIHC**	**LIRI-JP**	

NR1I3	0.978	0.978	
HNF4A	0.969	0.984	
RORC	0.935	0.888	
F2	0.975	0.967	
CREB3L3	0.856	0.876	

The knockout of *ETV7* produced the greatest decrease in growth rate in all three classes, as shown in Table 1. *ETV7* is a TF belonging to the ETS family, which is responsible for the development of different tissues as well as the progression of several cancers, such as HCC (Peeters et al., 1997; Matos et al., 2009). Due to its translocation function, the overexpression of *ETV7* has been associated with tumorigenic transformation and restriction of apoptosis by blocking the Mys-induced apoptosis pathway (Cardone et al., 2005; Carella et al., 2006; Federica et al., 2018). Accumulating experimental evidence indicates that *ETV7* also plays a significant role in the mTOR signaling pathway by assembling the mTOR3 complex, which can stimulate cell proliferation and is not sensitive to rapamycin, a common anti-tumor agent, unlike mTOR1/2 (Harwood et al., 2018). Therefore, *ETV7* depletion may cause the inactivation of mTOR3 and lead to tumor cell death after treatment.

*CTBP1* is a well-known cancer hallmark. The gene is linked with a pro-tumorigenic process and can affect the regulatory network (Blevins et al., 2017). It can bind to the C-terminus of the adenovirus protein E1A to promote cell proliferation and invasion (Hildebrand and Soriano, 2002). In addition, the characteristic elevated NADH level of cancer cells makes it possible for *CTBP1* to bind to NADP with a high affinity, thus triggering a conformational change that leads to hyper-activity of both tumorigenesis and tumor progression.

To explore the characteristics of TFs leading to low survival rate and poor prognosis, we selected TFs whose knockout only influenced samples in Class 2 of the TCGA-LIHC dataset. Five TFs were specific for Class 2 (threshold: ratio <0.98). Four of the five TFs were also associated with the lowest survival subgroup (Class 3) of the LIRI-JP dataset (the ratio of HNF4A somewhat exceeded the threshold), as shown in Table 1.

The knockout of *CREB3L3*, which was predicted to decrease the growth rate of tumor cells by over 15% but which had no effect on normal tissues, is reportedly activated with PPARα for lipid metabolism in liver-specific tissue (Vecchi et al., 2013). They both play important roles in the utilization of fatty acid for energy in a fasting state and in cell proliferation. Thus, it was not surprising that its absence was predicted to result in a decreased growth rate in tumor cells. The expression of *CREB3L3* is linked with restricted apoptosis, cell survival, and HBV-associated HCC development by regulating hepatic genes in the PI3K-Akt and AMPK signaling pathways. The alignment of *in-silico* analyses and biological knowledge suggests that *CREB3L3* is a potential therapeutic target, especially for advanced-stage HCC patients.

### Metabolic Genes in Cholesterol Biosynthesis Are Druggable Targets in HCC Treatment

We incorporated patient-specific models established by Uhle et al. (2017) to do metabolic analyses, including identification of metabolic genes essential for tumor cell growth and annotation of the specific reactions altered during tumor development. All 315 metabolic models were built to represent tumor growth. Using the genetic human metabolic model HMR2 and RNA-Seq expression data from TCGA-LIHC, a task-driven model reconstruction algorithm called tINIT was employed to construct all models.

We performed a single gene deletion simulation using a function provided in the COBRA Toolbox. The total gene number of each model ranged from 1,106 to 2,169. We first identified the essential genes in the three subtypes of TCGA-LIHC samples calculated by the NMF stratification strategy. We then collected genes that were essential in at least half of the samples in each class. Nineteen, 20, and 18 genes remained for Class 1, Class 2, and Class 3, respectively, after filtering those having no influence on tumor cell growth. Of these, the 18 genes found in Class 3 (relatively high OS rate) were are also found in the other two classes. *ACADSB* was shared by Class 1 and Class 2, and *CMPK1* was only identified in Class 2. We assessed prior knowledge about the therapeutic potential of these 20 genes in DrugBank^6^. The findings are summarized in Table 2.

**TABLE 2:** Lethal metabolic genes as potential targets and corresponding drugs in DrugBank.

Lethal gene	Target drug	Drug state	Brief description of drug
IDI1	Dimethylallyl diphosphate	Experimental	Unknown
SQLE	Ellagic acid	Investigational	Antioxidant and anti-proliferative/anti-cancer effects
FDFT1	TAK-475	Investigational	Target rate-limiting enzyme in the hepatic biosynthesis of cholesterol
CRAT	Levocarnitine	Approved	Treatment of primary systemic carnitine deficiency
EBP	Tamoxifen	Approved	Treatment of metastatic breast cancer
ACADSB	Isoleucine	Approved	Anti-proliferative effects useful in cancer therapy
	Valproic Acid	Approved	
SLC22A5	Levocarnitine	Approved	Treatment of primary systemic carnitine deficiency, affect fatty acid synthesis
HMGCR	Lovastatin	Approved	Lowering LDL cholesterol and triglycerides, hypercholesterolemia;
	Cerivastatin	Approved	Target rate-limiting enzyme in the hepatic biosynthesis of cholesterol
	Simvastatin	Approved	
	Atorvastatin	Approved	
	Rosuvastatin	Approved	
	Meglutol	Experimental	
CMPK1	Gemcitabine	Approved	Various advanced cancers
	Lamivudine	Approved	Treatment of HBV
	Sofosbuvir	Approved	Treatment of HCV
			Reduce incidence of HCC
MVK	Farnesyl thiopyrophosphate	Experimental	Unknown
HSD17B7	NADH	Approved	Treating Parkinson’s disease, chronic fatigue syndrome, Alzheimer’s disease and cardiovascular disease
NSDHL	NADH	Approved	Treating Parkinson’s disease, chronic fatigue syndrome, Alzheimer’s disease and cardiovascular disease
DHCR7	NADH	Approved	Treating Parkinson’s disease, chronic fatigue syndrome, Alzheimer’s disease and cardiovascular disease
ACACB	Soraphen A	Experimental	Anti-HCV viral activity
LSS	R048-8071	Experimental	Unknown
	Lanosterol	Experimental	
FDPS	Pamidronic acid	Approved	Treating severe hypercalcemia of malignancy
	Zoledronic acid	Approved	Treating Paget’s disease of bone
	Alendronic acid	Approved	Treating bone metastases from solid tumors
	Ibandronate	Approved, investigational	Treating osteolytic lesions of multiple myeloma
	Risedronic acid	Approved, investigational	Experimental drugs’ targets are still unknown
	ISOPENTENYL PYROPHOSPHATE	Experimental	
	Dimethylallyl Diphosphate	Experimental	
	Farnesyl diphosphate	Experimental	
	Geranyl Diphosphate	Experimental	
	Geranylgeranyl diphosphate	Experimental	
	Isopentyl Pyrophosphate	Experimental	
	Incadronic acid	Experimental	
CYP51A1	Levoketoconazole	Investigational	Treating fungal infections in immunocompromised and non-immunocompromised patients
	(S)-econazole	Experimental	Treating diabetes mellitus type 2.
	Miconazole	Approved, investigational, vet_approved	
	Itraconazole	Approved, investigational	
	Tioconazole	Approved	
PMVK	Unknown	Unknown	Unknown
MVD	Unknown	Unknown	Unknown
SC5D	Unknown	Unknown	Unknown

The DrugBank analysis identified 11 genes (*CRAT, EBP, ACADSB, CMPK1, SLC22A5, HMGCR, HSD17B7, NSDHL, DHCR7,FDPS*, and *CYP51A1*) that have already been targeted by approved drugs in the treatment of cancer or relative diseases. Six other genes have corresponding drugs being evaluated experimentally or investigationally. Both *CMPK1* and *ACADSB* seem to be vital to tumor cell growth in HCC models with a lower survival rate. These genes have been implicated as prognosis biomarkers associated with worse survival in multiple tumors for a long time (Ryu et al., 2011; Ohmine et al., 2015; Liu N.Q. et al., 2016; Zhou et al., 2017a,b; Zhang B. et al., 2019). *CMPK1* is also the target of three FDA approved cancer drugs (Gemcitabine, Lamivudine, and Sofosbuvir) for the treatment of diseases induced by a virus infection, such as HCC caused by HBV/hepatitis C virus. Li et al. (2019) recently reported that in Kaposi’s sarcoma, a common acquired-immunodeficiency-syndrome-related malignancy caused by infection of Kaposi’s sarcoma-associated herpesvirus, the invasiveness and motility of cells can be increased by overexpression of *CMPK*. This effect has also been validated by the knockout experiments carried out in cell lines. *FDPS* has been targeted by 11 drugs, among which five types of drugs are approved for mainly treating osteoporosis as well as bone metastases from solid tumors. *CYP51A1* has been the targets of three approved drugs, which are mainly used for treating fungal infections.

Among the 18 genes lethal in all three classes, 15 genes participate in cholesterol biosynthesis via the desmosterol (DESMOL) pathway, which is the dominant form of liver cholesterol biosynthesis (Song et al., 2005). The *HMGCR, MVK, PMVK, MVD*, and *IDI1* genes involving in the mevalonate pathway that converts acetyl-CoA to dimethylallyl pyrophosphate (DMAPP). The enzyme encoded by *FDPS* aids DMAPP in synthesizing farnesyl pyrophosphate (FAPP). *FDFT1* catalyzes the dimerization of two FAPP into squalene (SQNE). In the next step, *SQLE* and *LSS* play important rate-limiting roles in cholesterol biosynthesis by catalyzing the conversion of SQNE to lanosterol (LNSOL). LNSOL then goes through demethylation, oxidation, and reduction steps catalyzed by *CYP51A1*, *NSDHL*, and *HSD17B7* to form zymosterol (ZYMOL), the precursor in the DESMOL pathway. The *EBP, SC5D*, and *DHCR7* gene catalyze the conversion of ZYMOL to DESMOL. Finally, DESMOL is reduced by *DHCR24* to produce cholesterol. Knockout of any of these genes will disrupt cholesterol biosynthesis and lead to the depletion of cholesterol, which is disastrous for tumor cell growth.

There are only three predicted essential genes that have not been recorded in DrugBank. The high hit rate of drug targets (17/20) suggested that those three metabolic genes are potential targets and worthy of exploration in future studies. As mentioned above, *PMVK* and *MVD* are involved in the mevalonate pathway that converts acetyl-CoA to DMAPP. It has been reported that a key enzyme *HMGCR*, in the mevalonate pathway was confirmed to be closely related to cancer (Jiang et al., 2019). These three genes together help the transformation from Mevalonic acid to Isopentenyl diphosphate (IPP). *SC5D* catalyzes a dehydrogenation to introduce C5-6 double bond into lathosterol in cholesterol biosynthesis. Krakowiak et al. (2013) found that the mouse with *SC5D* disruption had elevated lathosterol, decreased cholesterol levels, and abnormal hedgehog signaling, which is considered to be related to tumorigenesis (Patrycja et al., 2003). Furthermore, *SC5D* regulates the enzyme converting lathosterol to 7-Dehydrocholesterol. And the downstream gene *DHCR7*, which converts 7-Dehydrocholesterol to cholesterol, has been targeted by drugs inhibiting HBV infection (Xiao et al., 2020). Therefore, although the three genes are currently not targets of existing drugs, they are all related to the main-effect pathway cholesterol biosynthesis and important for tumorigenesis of HCC.

### Enhancement of Glutathione and Fatty Acid Biosynthesis Are Important Metabolic Reprogramming Events Associated With Poor Prognosis

It is widely accepted that tumor cells reprogram metabolic pathways to enable unlimited cell proliferation, aggressive invasiveness, and restricted apoptosis. We investigated 1,329 reactions in all 315 patient-specific models to identify flux patterns and enzymes that differed between the poor prognosis subgroup (Class 2) and the other two classes. We conducted flux balance analysis with cell growth as the objective function to calculate the flux distribution for each patient, and selected candidate reactions having similar flux changes in over half samples of each subgroup. Four flux alteration patterns were evident. The first was from negative flux value in Class 1 and Class 3 to positive flux in Class 2. The second was from positive flux value in Class 1 and Class 3 to negative flux in Class 2. The third was from a non-zero flux in Class 1 and Class 3 to zero flux in Class 2. The fourth was from zero flux in Class 1 and Class 3 to non-zero flux in Class 2. The altered reactions, formulas, enzymes, and corresponding types of flux patterns are shown in Supplementary Table 4.

Two reactions simulated type 1 and type 2 flux change, respectively. According to these four reactions, the production of glutathione (GSH) was suspected to increase in Class 2 samples due to the enhancement of AKG biosynthesis and cysteine accumulation in the cytosol. GSH is a key member of the cell immune response system. The lack of GSH can easily lead to cell death. Several labs have confirmed its common occurrence in all cancers (Mehrmohamadi et al., 2014) and it is considered a potential therapeutic target. Additionally, loss of the enzyme catalyzing these reactions (encoded by *SLC25A11*) inhibits tumor cell growth in non-small cell lung cancer (Lee et al., 2019). Baulies et al. (2018) suggested that the overexpression of *SLC25A11* works as an adaptive mechanism of HCC to provide enough GSH for abundant cell growth, while *SLC25A11* induces the export of AKG to the cytosol to activate the mTOR pathway to promote cell growth and anabolism through egl-9 family hypoxia-inducible factors (EGLNs) (Villar et al., 2015).

Eleven reactions displayed no flux in Class 2 but a positive flux in Class 1 and 3 (type 3). Four of these reactions are part of porphyrin metabolism. The enzyme encoded by *UROD* is involved in this pathway and was recently identified as a potential anti-cancer target due to its ability to convert uroporphyrinogen to coproporphyrinogen (Yip et al., 2014). Another enzyme encoded by *ALAD* is overexpressed in breast cancer patients with a favorable clinical outcome. Its upregulation can suppress cell proliferation and invasion (Ge et al., 2017). In addition, a set of enzymes responsible for carnitine shuttling, which are encoded by *SLC22A1, SLC25A20, SLC25A29*, and *CPT2*, are downregulated in HCC tumor cells. These enzymes play rate-limiting roles in controlling fatty acid oxidation (Meihua et al., 2018). Their low expression has been significantly associated with worse patient survival (Heise et al., 2012) and differentiation state by impairing production of nitric oxide and the mTOR signaling pathway mediated by arginine. In some situations, this can lead to severe autophagy (Lifeng et al., 2016; Keshet and Erez, 2018).

Three reactions displayed non-zero flux in Class 2 but zero flux in Class 1 and 3 (type 4). These involved fatty acid activation responsible for providing adequate ATP and CoA for tumor cell growth; glycine, serine, threonine metabolism, which helps reduce reactive oxygen species pressure through the serine–glycine-one-carbon metabolic network during tumor metastasis (Amelio et al., 2014); and arginine/proline metabolism, which can regulate response to nutrient and oxygen deprivation in oncogenesis, thus avoiding cell apoptosis (Phang et al., 2015). Furthermore, exploration of enzymes revealed that *ACADSB* (which was also highlighted by previous analyses), *ACSL3*, and *ACSL4* regulate proteins that stimulate tumor cell proliferation, including p-AKT, LSD1, and β-catenin (Wu et al., 2015).

The altered reactions specific to Class2 samples promote tumor cell growth and decrease sensitivity towards normal apoptosis signals. Several key enzymes have already been implicated as biomarkers in cancers. Metabolic reprogramming accounting for poor prognosis also supports our stratification of the HCC patients.

### miRNAs Regulating Influential Genes for HCC Cell Proliferation

To investigate the interplay between regulation and metabolism of HCC further, we retrieved miRNAs regulating the influential genes highlighted in our previous analyses. These include the three common TFs that were influential in all three classes, the five overlapping TFs that specifically affected the lowest survival subgroup of TCGA-LIHC (Class 2) and LIRI-JP (Class 3), and the 20 metabolic genes revealed by single-gene deletion result (Supplementary Table 5). Evaluation of the MIRNET database identified the miRNAs functioning in liver tissue with higher connections to target TFs/genes. We found six miRNAs connected to the 28 genes of interest (Supplementary Table 5). Three of these were directly linked with HCC. MiR-124-3p and miR-1-3p have been reported to be downregulated in HCC patients compared to normal subjects (Lang and Ling, 2012; KöBerle et al., 2013). MiR-24-3p is involved in an HCC diagnosis panel because of its abnormal overexpression.

**FIGURE 5:**
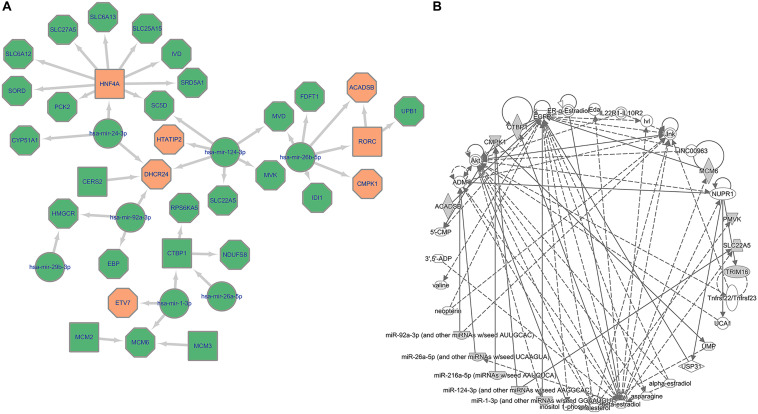
Multi-scale network exploration of HCC mechanism. **(A)** Core miRNA relation network of potential therapeutic targets. The octagon represents metabolic genes while the square represents TFs and the circle represents miRNAs; The orange color represents genes/TFs that have been experimentally validated as crucial genes in HCC tumor cells. **(B)** Biological connection of 34 genes (28 candidate genes and top 6 miRNAs).

The specific mechanism concerning how the loss-of-function or gain-of-function of these miRNAs contribute to tumorigenesis remains unclear. However, there are some experiment-based hypotheses. Figure 5A depicts the core network comprising miRNAs, TFs, and genes involved in HCC. The data will inform further studies in HCC development.

In particular, miRNA-124-3p appears to be the key miRNA during oncogenesis in many cancers (Murakami et al., 2006; Dai et al., 2009; Vlierberghe et al., 2010). Zheng et al. (2012) opined that miR-124-3p participates in reducing tumor cell motility and invasion by controlling epithelial–mesenchymal cell transition as well as cytoskeletal events through a cpG-island methylation (Furuta et al., 2010).

Zhang H. et al. (2019) suggested that miR-1-3p overexpression can inhibit cell proliferation and induce apoptosis by targeting the PI3K-Akt and mTOR pathways through ETV7. The downregulation of mir-24-3p can assist this process by deactivating the Fas receptor in the NOTCH pathway and inhibiting *HNF4A* to drive a feedback loop that leads to cancer-related inflammatory reaction (Salam et al., 2016). Additionally, Wang G. et al. (2017) and Chen et al. (2016) indicated that the regulatory impact of miR-24-3p includes an altered cell cycle by inducing p53 mutation as well as the avoidance of cell death by targeting the Fas receptor in the NOTCH pathway (Nicolas et al., 2003).

In addition, miR-26b-5p, which was connected to nine of the 28 genes, has been experimentally validated to be under-expressed in HCC patients with a worse prognosis. It can suppress tumor invasion as well as inducing apoptosis by targeting *SMAD1* (Wang et al., 2016), which is consistent with our conclusion about the *SMAD* gene. Three of the genes obtained by our integrated regulatory-metabolic analysis (*CMPK1, ACADSB*, and *RORC*) are directly regulated by miR-26b-5p. The fact that they are all specific genes for Class 2 (the class with the worst OS rate) substantiates the previous association.

Ingenuity Pathway Analysis (Krämer et al., 2013) of the 28 candidate genes and six top-connected miRNAs was performed to explore the biological connection among them. As shown in Figure 5B, *EGFR* was inferred and linked with our core gene set. *EGFR* is one of the most crucial genes responsible for cancer cell growth. Its overexpression can lead to unlimited cell proliferation, just like that in tumor cells. The gene is a potential therapeutic target in cancer therapy. Multiple FDA approved drugs, such as Gefitinib and Lapatinib, are effective in EGFR-related non-small-cell lung cancer and several other cancers (Rawluk and Waller, 2018; Voigtlaender et al., 2018).

## Discussion

### Integrated Regulatory-Metabolic Network Differences Between HCC and Normal Liver Cells

The curated information linking the reactions of genes and proteins in GEMs has enabled the identification of many potential disease-related biomarkers by metabolic analyses. The interconnectedness between metabolism and regulation permits the integration of regulatory with metabolic models, which in turn allows the more precise description of the phenotypic impact of mutations and environmental perturbations. This integration has proven effective in model organisms, including *S. cerevisiae* and *E. coli*, but has not yet been applied to the study of human diseases.

Here we leveraged the mechanistic modeling of transcriptional regulatory network and metabolic network for HCC study, by extensively improving our IDREAM framework. We used two different approaches to construct transcriptional regulatory networks for HCC and normal liver tissue samples. Through topology analysis, *NME2*, and *NFBIKA* were implicated as tumor suppressor TFs because of their absence in a tumor regulatory network and high connectivity in a non-tumor network. We integrated the regulatory networks with a human liver metabolic model, and compared the effects of TFs on cell growth in tumor and normal models. TFs that only reduced the growth of tumor cells were predicted to be potential targets. These included the *SMAD2, HEY2, ELK1*, and *CREB3L3* genes.

### Three Subtypes of HCC Samples Demonstrate Significantly Different Prognosis

By allocating TCGA-LIHC samples using pre-filtered 3,492 genes, we defined three patient subgroups distinguished by the OS rate. Patients in Class 2 displayed the worst survival. We identified three essential TFs for HCC tumor cell growth that were common in all three groups. Among these, *ETV7* displayed the greatest impact, decreasing cell growth rate by approximately 88% in Class 2. *ETV7* is a TF belonging to the ETS family. It is responsible for the progression of several cancers, including HCC. Because of its translocation function, the overexpression of *ETV7* has been associated with tumorigenic transformation and restricted apoptosis by blocking the Mys-induced apoptosis pathway. There is growing evidence of a significant role of *ETV7* in the mTOR signaling pathway, which involves the assembly of the mTOR3 complex to stimulate cell proliferation and prevent cell damage by rapamycin, a common anti-tumor agent.

In addition, we identified potential TFs related to poor prognosis based on the simulated knockouts of five TFs, which were predicted to specifically affect patients in Class 2. Among these five TFs, *CREB3L3* was also predicted as being influential for advanced-stage HCC samples by the TF knockout simulation in the generic integrated regulatory-metabolic model. It has been reported that the expression of *CREB3L3* is linked with cell survival and HBV-associated HCC development by regulating hepatic genes in the PI3K-Akt and AMPK signaling pathways (Vecchi et al., 2013).

The poor prognosis group (Class 2) also exhibited a specific pattern of altered metabolism. Flux alterations in Class 2 samples included the accumulation of both AKG and cysteine, which indicated the over-production of GSH, a key member of the cellular immune response system that improves cell proliferation and avoids apoptosis. Besides the biosynthesis of fatty acids, mTOR signaling was also hyper-activated, and pathways that included those of glycine, serine, and threonine metabolism reduce reactive oxygen species stress during tumor homeostasis.

We used the same stratification strategy for the LIRI-JP dataset. Survival outcomes likewise displayed significant differences among the subgroups. The predicted outcomes of TFs affecting the lowest survival subgroup were consistent with that of the TCGA-LIHC dataset.

### Key Metabolic Genes in Cholesterol Biosynthesis Identified by Patient-Specific Models Are Potential Targets

The metabolic analyses based on patient-specific models revealed 20 metabolic genes with important roles in HCC tumor cell growth by participating in the cholesterol biosynthesis pathway. Recent research uncovered that cholesterol biosynthesis supports the growth of hepatocarcinoma lesions depleted of fatty acid synthase, concomitant targeting de novo lipogenesis and cholesterol biosynthesis are highly detrimental for the growth of human HCC cells (Che et al., 2019)

According to DrugBank, eleven genes have already been therapeutically targeted in various cancers or cancer-related diseases, and six other genes have corresponding drugs being evaluated experimentally or investigationally. Although the remaining three genes, *PMVK*, *MVD*, and *SC5D* are currently not targets of existing drugs, they are all related to the main-effect pathway cholesterol biosynthesis and important for tumorigenesis of HCC, which might become novel potential therapeutic targets and worthy of exploration in future studies. We further found that *ACADSB* and *CMPK1* appeared to be specifically essential in Class 2. These two genes could be associated with poor prognosis and may be the targets for the treatment of more serious HCC patients.

### Multi-Scale Regulatory-Metabolic Network Reveals a Critical Mechanism of HCC Cell Proliferation

In addition to the integration of transcriptional regulation with metabolism, it is well known that dysregulated miRNAs also played an important regulatory role in tumorigenesis. We incorporated the miRNAs regulating the identified influential TFs and metabolic genes generated from an integrated transcriptional regulatory-metabolic network model. Based on the highlighted genes (total of 28 key genes), we predicted miRNAs regulating these candidates using MIRNET. Three miRNAs (miR-124-3p, miR-1-3p, and miR-24-3p) have been described as important factors associated with HCC tumorigenesis and function in established cancer-related pathways, including NOTCH, PI3K-Akt, and mTOR. We illustrated the core network of HCC cell proliferation involving interactions between miRNAs-TFs, miRNAs-Targets, and TFs-Targets (Figure 5A), and emphasized the targets that were highlighted in the combined analyses. In general, the inhibition of miRNAs on overexpressed genes in HCC were consistent with their validated function such as suppressing tumorigenesis. The findings suggest potential mechanisms associating the key genes predicted from our regulatory-metabolic network analysis with cancer cell growth outcomes. Notably, the direct regulation of miR-26b-3p on *ACADSB* and *CMPK1* provides experimental evidence to support the idea that these two metabolic genes are linked with lower OS in HCC. Moreover, the biological connection inferred by the Ingenuity Pathway Analysis indicated these highlighted genes are closely connected to *EGFR*, which plays a significant role in cancer cell proliferation, providing evidence for our comprehensive analyses.

## Data Availability Statement

The expression data that support the findings of this study are available in GEO Datasets with the identifier (10.18632/oncotarget.8927) (Liu G. et al., 2016). The expression and clinical data that support the findings of this study are available in TCGA Database, LIHC section (https://portal.gdc.cancer.gov/). The expression and clinical data that support the findings of this study are available in ICGC database, JIRI-JP section (https://icgc.org/). The genome-scale metabolic liver model that support the findings of this study is available in HMA database (https://metabolicatlas.org/gems/repository). The patient-specific metabolic models that support the findings of this study are available in BioModels Database (https://www.ebi.ac.uk/biomodels/) with the identifier (doi: 10.1126/science.aan2507) (Uhle et al., 2017).

## Author Contributions

RS was responsible for the data gathering, model integration, metabolic analysis, and manuscript writing. YX was responsible for regulatory network inferring, statistical analysis, network topology analysis, and literature mining. HZ was responsible for the TCGA and ICGC RNA-Seq the data gathering and the data preprocessing. QY was responsible for the IPA analysis. KW was responsible for the survival analysis. YS involved in design and management of the project. ZW was responsible for the design of the integrated model, explanation of results, and manuscript writing. All authors contributed to the article and approved the submitted version.

## Conflict of Interest

The authors declare that the research was conducted in the absence of any commercial or financial relationships that could be construed as a potential conflict of interest.

## Footnotes

1 https://metabolicatlas.org/gems/repository

2 https://www.ebi.ac.uk/biomodels/

3 http://regulatorycircuits.org/

4 http://www.regnetworkweb.org/

5 https://david.ncifcrf.gov/

6 https://www.drugbank.ca/

## Supplementary Material

Supplementary File 1 The scripts for the integrated regulatory-metabolic model construction and prediction of TF/Gene knockout effects on cancer cell growth.

Supplementary Table 1 Composition of normal/HCC liver model: The regulatory network of normal/HCC liver and the composition of integrated models.

Supplementary Table 2 TFs affecting normal/HCC liver cell growth and corresponding growth ratio after TFs knocking down.

Supplementary Table 3 Differentially expressed genes in Class2 compared to Class 1 and Class 3 in TCGA-LIHC samples.

Supplementary Table 4 Altered metabolic reactions in Class2 compared to Class 1 and Class 3 in TCGA-LIHC samples.

Supplementary Table 5 28 core genes filtered according to our research.
