# Supplementary material for: Mechanistic Modeling of Gene Regulation and Metabolism Identifies Potential Targets for Hepatocellular Carcinoma
Source: Front Genet. 2020 Dec 23;11:595242. doi: 10.3389/fgene.2020.595242 (PMC7786279; doi:10.3389/fgene.2020.595242)
Supplement: Supplementary File 1 — The scripts for the integrated regulatory-metabolic model construction and prediction of TF/Gene knockout effects on cancer cell growth. [file Data_Sheet_1.PDF]

#The procedure is the same for Tumor and Normal sample data, just replace the corresponding file

#######Part 1. Filter expression data of genes for constructing regulatory network (PYTHON)#######

```
import pandas as pd
data=pd.read_csv('original_GSE77341_exp_data') #row-names are geneID and column-names are sampleID
gene=pd.read_csv('totalgene')   #1-column file with column name'GENE'
result_data=pd.DataFrame(columns=data.columns)
gene_li=list(gene['GENE'])
exp_li=list(data['gene_id'])
c=0
for item in gene_li:
	try:
		ind=exp_li.index(item)
		result_data=result_data.append(data.loc[ind,])
	except:
		pass
result_data.to_csv('gene_exp_data')
```

#######Run MERLIN(TERMINAL)#######

```
#After convert 'gene_exp_data' into MERLIN format('MERLINmettf_exp_data') and prepare TF list file('MERLINtflist'), details can be found in MERLIN paper
#Because parameter 'v' is 5, so we will get 5 independent results named 'prediction_1' to 'prediction_5' in export_path
./merlin -d 'input_path/MERLINmettf_exp_data' -o 'export_path' -l 'input_path/MERLINtflist' -v 5 -h 0.6 -k 300 -p 5 -r 4
```

#######Select regulations exsiting in at least 3 of 5 MERLIN results(PYTHON)#######

```
predict_1=pd.read_csv('prediction_1',sep='\t',header=None,usecols=[0,1])
predict_2=pd.read_csv('prediction_2',sep='\t',header=None,usecols=[0,1])
predict_3=pd.read_csv('prediction_3',sep='\t',header=None,usecols=[0,1])
predict_4=pd.read_csv('prediction_4',sep='\t',header=None,usecols=[0,1])
predict_5=pd.read_csv('prediction_5',sep='\t',header=None,usecols=[0,1])
temp_result=pd.concat([predict_1,predict_2,predict_3,predict_4,predict_5],axis=0)
dic_collect={}
dic_count={}
regulatory_pair=[]
count=0
for row in range(len(temp_result)):
	regulatory_pair.append('.'.join(list(temp_result.iloc[row,:])))
for item in range(len(regulatory_pair)):
	if regulatory_pair[item] in dic_collect:
```

```
            dic_collect[regulatory_pair[item]]+=1
        else:
            dic_collect[regulatory_pair[item]]=1
for key,value in dic_collect.items():
        if value>=3:
            dic_count[key]=value
result=pd.DataFrame(dic_count,index=[0])
result.describe
result_T=result.T
final_result['TF'],final_result['TARGET']=
result_T.iloc[:,0].str.split('.').str
final_resul.to_csv('MERLIN_regulatory_result')

#######Run CMIP(TERMINAL)#######
##After convert '3492gene_exp_data' into CMIP format('CMIPmettf_exp_data'),
details can be found in CMIP paper
./CMIP 'input_path/CMIPmettf_exp_data' no 'export_path' 1

#######Re-build CMIP result into TF-TAR format#######
CMIP=pd.read_csv('Relation')
CMIP.columns=['tf','target','Rela']
temp_CMIP=CMIP[CMIP['Rela']==1]
metgene=pd.read_csv('metgenelist')
tfgene=pd.read_csv('tflist')
target=list(metgene['METNAME'])
tf=list(tfgene['TFNAME'])
result_CMIP=pd.DataFrame(columns=temp_CMIP.columns)
count=0
for i in range(len(temp_CMIP['target'])):  #keep 'TF-TAR' or 'TAR-TF' relations
and remove 'TF-TF' or 'TAR-TAR' relations
        if temp_CMIP.ix[i,'target'] in target:
            if temp_CMIP.ix[i,'tf'] in tf:
                result_CMIP.loc[count]=temp_CMIP.ix[i,['tf','target']]
                count+=1
        if temp_CMIP.ix[i,'tf'] in target:
            if temp_CMIP.ix[i,'target'] in tf:
                result_CMIP.loc[count]=temp_CMIP.ix[i,['target','tf']]
                count+=1
result_CMIP.to_csv('CMIP_regulatory_result',index=False)

#######Combine MERLIN results with CMIP results#######
MERLIN_result=pd.read_csv('MERLIN_regulatory_result')
CMIP_result=pd.read_csv('CMIP_regulatory_result')
pre_result=pd.cancat([CMIP_result,MERLIN_result],axis=0)
dic_combine={}
```

```
merge_pair=[]
count=0
for row in range(len(pre_result)):
	merge_pair.append('.'.join(list(pre_result.iloc[row,:])))
for item in range(len(merge_pair)):   #Mark the relations inferred by both
algorithms as 1, and the rest as 0 for IDREAM integration
	if merge_pair[item] in dic_combine:
		dic_combine[merge_pair[item]]+=1
	else:
		dic_combine[merge_pair[item]]=0
result_temp=pd.DataFrame(dic_combine,index=[0])
result_temp.describe
result_temp_T=result_temp.T
merge_result['TF'],merge_result['TARGET']=
result_temp_T.iloc[:,0].str.split('.').str
merge_result['confidence']=result_temp_T.iloc[:,1]
merge_result.to_csv('Combine_MERLIN_CMIP_result')

#######Part 2: Construction of the integrated regulatory-metabolic network model
using MATLAB#######
initCobraToolbox;
changeCobraSolver('gurobi5','LP')
exp_data=importdata('3492gene_exp_data');
regulatory_data=importdata('Combine_MERLIN_CMIP_result');
load('Livermodel');
[f,f_ko,v,v_ko,status1,lostrxns,probtfgeneb]=promv2(Livermodel,3492gene_exp_da
ta.data,3492gene_exp_data.textdata,regulatory_data.textdata(:,1),regulatory_da
ta.textdata(:,2),regulatory_data.data,regulatory_data.data);

#######function 'promv2' source code (MATLAB)#######
function [f,v,status1,lostxns] =
promv2(model,expression,expressionid,regulator,targets,litevidence,prob_prior)
%%%%%%%%%%%%%%%%%%%%%%%%%%%%%%%%%%%%%
%% [f,v,status1,lostxns] =
promv2(model,expression,expressionid,regulator,targets,litevidence,prob_prior)
;
% The PROM algorithm predicts the growth phenotype and the flux response
% after transcriptional perturbation, given a metabolic and regulatory
% network.
% INPUTS
% Model is the metabolic model obtained from COBRA toolbox through readcbmodel
command
%
% Gene expression data - rows - genes,columns - conditions; no need to normalize
or impute
```

```
%
% Expressionid - an array of identifiers for each row/gene should be included
%
% regulatory network - format - cell array of regulators and matching target genes
% example   Regulator = {'RegA'; 'RegB' ; 'RegC'};  Targets =
% {'GeneA';'GeneB';'GeneC'}
% note that the names or identifiers used in the regulatory data should
% match the names/ids given for gene expression data
%
%OPTIONAL - litevidence & prob_prior -> these should have the same length
%as the regulator/target arrays;
% high confidence interactions (not necessarily based on literature) should be
flagged as one in litevidence
%array and the rest should be set as 0.
% Prob_prior should be set values between 0 and 1 for those interactions with
litevidence. ( other values in
%the array would be ignored)
%
% OUTPUT - the algorithm gives the growth rate (f) and flux response (v) after knock
out of all
% the regulators in the regulatory model; status is the glpk solver status
% the status should be 5 for glpk; if it is not then check solver error log
% lostxns gives the interactions that could not be quantified based on the
% threshold set for binarization
% the program would shoot a warning if the threshold chosen is bad. The
% default value (0.2 - 0.4) should work for most cases
%

%%%%%%%%%%%%%%%%%%%%%%%%%%%%%%%%%%%%%%%%%%%%%%%%%%

%% INPUT HANDLING
%===========================================================
if nargin == 5, litevidence = [];prob_prior = [];
elseif (nargin < 5) || (nargin == 6),
    error('Incorrect number of input arguments to %s',mfilename)
end
%===========================================================
%SOME BASIC INITIALIZATION
%===========================================================
disp('initializing data')
regulated = targets;
[tfnames,b,m] = unique(regulator);
weights = model.c; stoic  = model.S; S = model.S; ctype =
repmat('S',size(model.b));lbff = model.lb; ubff = model.ub;dxdt = model.b;
param.msglev = 1;
```

```
%%%%%%%%%%%%%%%%%%%%%%%%%%%%%%%%%%%%%%%%%%%%%%%%%%%%%%%%%%%%
[u,v] = find(model.rxnGeneMat);
% u - reaction; v - genes
% finding rxn position
rxnpos = u;
genelist = v;
clear u v

% I need to find the reactions that each gene has influence on
%%%%%%%%%%%%%%%%%%%%%%%%%%%%%%%%%%%%%%%%%%%%%%%%%%%%%%%%%%%%%%%%%%%%
bnumsinexpsn = expressionid;
litevidence = logical(litevidence);

%%%%%%%%%%%%%%%%%%%%%%%%%%%%%%%%%%%%%%%%%%%%%%%
scou = 1;
%% new additions
lbg = model.lb; ubg = model.ub;
a1 = [S,zeros(size(S,1),length(ubg)),zeros(size(S,1),length(ubg))];
a2 = sparse([eye(length(ubg)), eye(length(ubg)),zeros(length(ubg))]);
a3 = sparse([eye(length(ubg)), zeros(length(ubg)),-eye(length(ubg))]);
A = [a1;a2;a3];
weights11 = [weights;zeros(2*length(lbg),1)];
weights00 = [weights;zeros(2*length(lbg),1)];
lb11 = [-1000*ones(length(lbg),1);zeros(length(lbg),1);zeros(length(lbg),1)];
ub11 = [1000*ones(length(lbg),1);zeros(length(lbg),1);zeros(length(lbg),1)];
dxdt0 = [zeros(size(S,1),1);lbg;ubg];
ctype1 =
[repmat('S',size(S,1),1);repmat('L',size(lbg,1),1);repmat('U',size(lbg,1),1)];
[v0,f0] = glpk(-weights11,A,dxdt0,lb11,ub11,ctype1);

%%%%%%%%%%%%%%%%%%%%%%%%%%%%%%%%%%%%%%%%%%%%%%%
%% Find Probabilities using a Global Threshold
%%%%%%%%%%%%%%%%%%%%%%%%%%%%%%%%%%%%%%%%%%%%%%%
tic
lost_xn = false(size(regulated));

disp('finding probabilities')
cou = 1;cou1 = 1;cou3 = 1;
%data= knnimpute(datbackup);
data = expression;
data = knnimpute(data);
data = quantilenorm(data);
data1 = data;
```

```
%kappavals = [0,0.0001,0.001,0.05,0.1,0.25,0.33,0.5,0.75,1];
%datathreshvals = [0,0.01,0.05,0.1,0.2,0.25,0.33,0.4,0.5,0.75,1];
%datathresh = quantile(data(:),datathreshvals(scou));
datathresh = quantile(data(:),0.33);
if datathresh < 0,
    data(data>=datathresh) = 1;
    data(data < datathresh) = 0;
else
    data(data < datathresh) = 0;
    data(data>=datathresh) = 1;
end

for  i = 1:length(regulated)
    k = find(ismember(bnumsinexpsn,regulated(i)));
    l = find(ismember(bnumsinexpsn,regulator(i)));
    if ~isempty(k) & ~isempty(l)
        te = data1(k,:);
        te1 = data1(l,:);

        tec = data(k,:);

        tec1 = data(l,:);

        cou1 = cou1 + 1;
        try kstest2(te(tec1 == 1),te(tec1== 0));

            if  (kstest2(te(tec1 == 1),te(tec1== 0)) == 1),

                prob1 = sum(tec(tec1 == 0))/length(tec(tec1==0));
                probtfgene(i) = prob1;
                cou = cou + 1;
                %   this formula also gives the same answer  - (sum(~tec1(tec ==
1))/length(tec1(tec==1))) * (sum(tec)/sum(~tec1))

            else

                probtfgene(i) = 1;  % no effect

            end

        catch ERRLG    % cant be estimated from microarray ; if it has strong
evidence, consider setting this to zero
            probtfgene(i) = 1;
            lost_xn(i) = 1;
```

```
        end
    else
        probtfgene(i) = 1;
                    lost_xn(i) = 1;
    end
end

probtfgene = probtfgene(:);
toc
if (sum(lost_xn) > 0.75*length(probtfgene))   % check if there is problem with binarizartion.. usually there ll be some interactions that'd be missed, but if most of it (like >75% ) are...
    % missed then its time to change the threshold
   datathreshflag = 1;
   disp('change binarization threshold')
else
    datathreshflag = 0;
end

if ~isempty(litevidence)
    probtfgene(litevidence) = prob_prior(litevidence);  % u could set those interactions that u think have strong literature evidence to have predefined
end                                                            % probabilities

toc
```

#######Part 3. Prediction the effects on cell growth by each TF/Gene knockout (MATLAB)#######

```
%%%%%%%%%%%%%%%%%%%%%%%%%%%%%%%%%%%%%%%%%%%%%%%%
%%%%%%%%%%%%%%%%%%%%%%%%%%%%%%%%%%%%%%%%%%%%%%%%%%
disp('running PROM')
count = 1; thresh = 10^(-6); mthresh = 10^(-3);
allgenes = [model.genes;tfnames];
[ir,posgenelist] = ismember(regulated,model.genes);
lbf = lbff; ubf = ubff;
[v,f] = glpk(-weights,stoic,dxdt,lbf,ubf,ctype);
% kapavals = [0,0.0001,0.001,0.05,0.1,0.25,0.33,0.5,1,5,10];
%kappa = kapavals(scou);
 kappa = 1;
%%%%%%%%%%%%%%%%%%%%%%%%%%%%%%%%%%%%%%%%%%%%%%%%
hw = waitbar(0);
% knock out the genes
bnumstobekoed= tfnames;   % bnumstobekoed -  gives the geneids of the genes to be knocked out - by default it knocksout all the tfs in the model one by one
vm = zeros(size(v));
```

```
% bnumstobekoed = [tfs_that_are_off;tf_that u want to knockout];
for ci = 1:length(bnumstobekoed)
    lbg = lbf; ubg = ubf;
    lb11 =
[-1000*ones(length(lbg),1);zeros(length(lbg),1);zeros(length(lbg),1)];
    ub11 =
[1000*ones(length(lbg),1);zeros(length(lbg),1);zeros(length(lbg),1)];
    % check if its a metabolic or regulatory gene or both

    if any(strcmpi(model.genes,bnumstobekoed(ci)))
        temppos = rxnpos(genelist ==
find(strcmp(model.genes,bnumstobekoed(ci))));
        for jj = 1:length(temppos)
            if model.rev(temppos(jj))
                lbg(temppos) = -thresh;
                ubg(temppos) = thresh;
            else
                lbg(temppos) = -thresh;
            end
        end

    end

    [v1,fk(ci)]  = glpk(-weights,S,dxdt,lbg,ubg,ctype);

    if any(ismember(tfnames,bnumstobekoed(ci))),


        tfstate = logical(zeros(size(tfnames)));
        tfstate(find(ismember(tfnames,bnumstobekoed(ci)))) = 1;
        k = find(ismember(regulator,tfnames(tfstate)));
        tempgene = regulated(k);
        tempgeneprobs = probtfgene(k);
    %    k(tempgeneprobs == 1) = '';
        tempgene = regulated(k);
        tempgeneprobs = probtfgene(k);
        tempgenepos = posgenelist(k);
        temprxnpos = rxnpos(ismember(genelist,tempgenepos));
        %%%%%%%%%%%%%%%%%%%%%%%%%%%%%%%%%%%%%%%%%%%%%%%%%%%%%%%%%%%%%%%%
%%%%%%
        % this section is for gene-protein-reaction relationship
        x = true(size(model.genes));
        [isInModel,geneInd] = ismember(tempgene,model.genes);
        x(geneInd) = false;
```

```
        constrainRxn = false(length(temprxnpos),1);
        % Figure out if any of the reaction states is changed
        for j = 1:length(temprxnpos)
            if (~eval(model.rules{temprxnpos(j)}))
                constrainRxn(j) = true;
            end
        end
        % Constrain flux through the reactions associated with these genes

        %%%%%%%%%%%%%%%%%%%%%%%%%%%%%%%%%%%%%%%%%%%%%%%%%%%%%%%%%%%%%%%%%%%%%%%%
%%%%%%%%%%%%%%%%%%%%%%%%
        tempgeneprobs(tempgenepos == 0)  = '';
        tempgenepos(tempgenepos == 0)  = '';
        % temprxnpos has the rxns that are going to be affected by this tf
        % krxnpos are the rxns that will be affected by this target gene alone.
        % we loop around all the genes.



        for l = 1:length(tempgenepos)
            if ~isnan(tempgeneprobs(l))
                krxnpos =
ismember(temprxnpos,rxnpos(ismember(genelist,tempgenepos(l))));
                for m = 1:length(temprxnpos)
                    if krxnpos(m)
                        tempsubsysmid = 0;

                        if constrainRxn(m)
                            if (tempgeneprobs(l) < 1)    % if its 1 no use in
changing the bounds - might as well save time
                                if (tempgeneprobs(l) ~= 0)   % if its zero no
point in estimating vm again - saves time.. but cant include in the above statement
coz u have to change the bounds
                                    %if v(temprxnpos(m))
                                    if ~vm(temprxnpos(m))    % done to save time
- if estimated already use it
                                        weights1 = weights; lbv = lbf; ubv = ubf;
                                        grwthpos = find(weights == 1);
                                        lbv(grwthpos) = v(grwthpos);
                                        weights1(temprxnpos(m)) = -1;
                                        [v11,fva1]  =
glpk(-weights1,S,dxdt,lbv,ubv,ctype);
                                        weights1(temprxnpos(m)) = 1;
                                        [v12,fva2]  =
glpk(-weights1,S,dxdt,lbv,ubv,ctype);
```

```
                                if  v(temprxnpos(m)) < 0

                                    vm(temprxnpos(m)) =
min([v11(temprxnpos(m)),v12(temprxnpos(m)),v(temprxnpos(m))]);
                                elseif v(temprxnpos(m)) > 0
                                    vm(temprxnpos(m)) =
max([v11(temprxnpos(m)),v12(temprxnpos(m)),v(temprxnpos(m))]);
                                else
                                    vm(temprxnpos(m)) =
max([abs(v11(temprxnpos(m))),abs(v12(temprxnpos(m))),abs(v(temprxnpos(m)))]);
                                end


                            end
                            %end
                        end


                        xx =  ( vm(temprxnpos(m))*(1 - exp(-
tempsubsysmid) ) + ( vm(temprxnpos(m))*tempgeneprobs(l)*exp(-tempsubsysmid)));
                        if  v(temprxnpos(m)) < 0

                            tem =
max([lbf(temprxnpos(m)),xx,lbg(temprxnpos(m))]);  %make sure we arent violating
the original bounds; also get the lowest value if there were multiple modifications
for the rxn
                            lbg(temprxnpos(m)) =
min([tem,-thresh]);   % prevents the solver from crashing
                            ub11(1*length(ubg) + temprxnpos(m)) = 1000;
                            weights11(1*length(ubg) + temprxnpos(m)) =
(-1*kappa/abs(vm(temprxnpos(m))))*abs(f0);   % v0 f0 are the wild type values..
                            vv = max([abs(vm(temprxnpos(m))),mthresh]);
                            weights11(1*length(ubg) + temprxnpos(m)) =
min([(kappa*(-1)*abs(f0))/abs(vv), weights11(1*length(ubg) + temprxnpos(m)) ]);
                        elseif v(temprxnpos(m)) > 0
                            tem =
min([xx,ubf(temprxnpos(m)),ubg(temprxnpos(m))]);
                            ubg(temprxnpos(m)) = max(tem,thresh);
                            ub11(2*length(ubg) + temprxnpos(m)) = 1000;
                            vv = max([abs(vm(temprxnpos(m))),mthresh]);
                            weights11(2*length(ubg) + temprxnpos(m)) =
min([(kappa*(-1)*abs(f0))/abs(vv), weights11(2*length(ubg) +
temprxnpos(m)) ]);  % new weights based on kappa, normalized with growth rate
                        end
```

```
                        end
                    end
                end
            end
        end
    end
end

dxdt0 = [zeros(size(S,1),1);lbg;ubg];
[v00(ci,:),f00(ci),status1(ci)] =
glpk(-weights11,A,dxdt0,lb11,ub11,ctype1);
%%%%%%%%%%%%%%%%%%%%%%%%%%%%%%%%%%%%%%%%%%%%%%%%%%%%
coun  = 1;        lbh = lbg; ubh  = ubg;

while ((status1(ci) == 105) || (v00(ci,1028) < 0))
    lbh(lbh ~= lbf) = lbh(lbh ~= lbf) - 1E-3;
    ubh(ubh ~= ubf) = ubh(ubh ~= ubf) + 1E-3;
    dxdt0 = [zeros(size(S,1),1);lbh;ubh];
    [v00(ci,:),f00(ci),status1(ci)] =
glpk(-weights11,A,dxdt0,lb11,ub11,ctype1);
    coun = coun + 1;
    if (coun > 100),
        % if its impossible to estimate, then
        % check the unweighted g.r and the one with max weight prom -
        % if very less difference use it - warn the user about
        % the problem at the iteration number - ci;
        [v3,f3,status3] = glpk(-weights,S,dxdt,lbg,ubg,ctype);
        [v30,f30,status30] = glpk(-weights00,A,dxdt0,lb11,ub11,ctype1);
        if abs((f3-f30)/abs(f3)) < 0.1
            f00(ci) = f3;

        else
            disp(' problem in'); disp(ci);break;  % if that doesnt work,
display a warning
        end

        disp('check'); disp(ci); break;

    end
end
%%%%%%%%%%%%%%%%%%%%%%%%%%%%%%%%%%%%%%%%%%%%%%%%%%%%%%%%%%%%

lbg_st(ci,:) = lbg;
ubg_st(ci,:) = ubg;
```

```
    lb_st(ci,:) = lbff;
    ub_st(ci,:) = ubff;

        [v2(ci,:),f1(ci),status] = glpk(-weights,S,dxdt,lbg,ubg,ctype);


    ktime = toc;
    waitpar = [num2str(ceil(ci/length(tfnames)*100)),'% complete. Time 
taken:',num2str(ceil(ktime)),' secs'];
    waitbar(ci/length(tfnames),hw,waitpar);


    ff00(scou,ci) = v00(ci,1028);


    %%%%%%%%%%%%%%%%%%%%%%%%%%%%
        if datathreshflag
            if all(lost_xn(k))   % if none of the probabilities of a gene can be 
estimated, then ->
                v00(ci,:) = NaN;
                f1(ci) = NaN;
                v2(ci,:) = NaN;
                f00(ci) = NaN;
                %break;
            end
        end

%%%%%%%%%%%%%%%%%%%%%%%%%%%%%%%

    clear tempgenepos tempgeneprobs temprxnpos k

end

f_ko = -f1';
v_ko = v2;

f00_ko(:,scou) = v00(:,1028);
v00_ko = v00;

f = f00_ko; v = v00_ko;


lostxns(:,scou) = lost_xn;
end
```
